# Considerations and implications of current *in vitro* model systems to study optic nerve head cellular mechanobiology

**DOI:** 10.3389/fcell.2025.1699793

**Published:** 2025-10-24

**Authors:** Ana N. Strat, Preethi S. Ganapathy

**Affiliations:** ^1^ Department of Ophthalmology and Visual Sciences, SUNY Upstate Medical University, Syracuse, NY, United States; ^2^ Department of Neuroscience and Physiology, SUNY Upstate Medical University, Syracuse, NY, United States; ^3^ BioInspired Institute, Syracuse University, Syracuse, NY, United States

**Keywords:** compression, tension, bioengineering, glaucoma, hydrogels, stretch, astrocytes, lamina cribrosa cells

## Abstract

The optic nerve head (ONH) is a primary site of biomechanical strain within the eye. Here, tensile, compressive and shear forces can impact both physiologic function and pathologic dysfunction of the tissue. Under healthy conditions, the ONH can withstand dynamic fluctuations in intraocular pressure (IOP), which is the main driver of biomechanical strain. In glaucoma, pathologic biomechanical strains lead to disrupted cellular homeostasis, increased ECM remodeling and fibrosis, loss of retinal ganglion cells (RGCs), and irreversible blindness. *In vitro* model systems are increasingly used to dissect the mechanisms by which ONH cells respond to biomechanical strain (i.e., their mechanobiology). This review aims to present the current state of these *in vitro* model systems. Traditional two-dimensional (2D) stretch systems have provided important insights into ONH cellular mechanobiology. More recent 3D *in vitro* models include hydrogel-based systems that incorporate natural or synthetic ECM polymers that support development of cell morphology and ECM interactions similar to those observed *in vivo*; these models permit application of compressive and tensile biomechanical strain. This review also discusses ONH biomechanical strains as they pertain to glaucoma pathophysiology, the ONH cell types implicated in pathologic mechanobiology, and the experimental parameters generally used in current *in vitro* systems. Ultimately, refinements of these *in vitro* model systems can provide crucial mechanistic insights into cellular mechanodysfunction and potential therapeutic targets for glaucoma.

## Introduction

The optic nerve head (ONH) biomechanical environment is highly dynamic, and in healthy states, ONH tissues are able to withstand wide ranges of variable intensities and frequencies of biomechanical strain (Jin et al., 2020; Turner et al., 2019). This indicates that the cells within these regions can compensate for these physiologic changes in biomechanical strain. However, in pathologic states, local cells cannot meet the demands of excess biomechanical strains, resulting in tissue dysfunction (Burgoyne et al., 2005; Stowell et al., 2017). The pathognomonic disease of excess biomechanical strains on the ONH is glaucoma (Downs, Roberts, and Burgoyne, 2008; Burgoyne et al., 2005), wherein the tissue is subjected to increasing degrees of tensile, compressive, and shear biomechanical strains. Much of our understanding of cellular pathophysiology in glaucoma comes from acute, subacute, or chronic *in vivo* or *ex vivo* models of intraocular pressure elevation. While extremely informative, such models are not easily able to tease apart contributions of specific cell types to overall tissue behavior.

ONH cellular dysfunction in glaucoma is thought to be multifactorial, with oxidative stress, excitotoxicity, metabolic insufficiency, glial activation, inflammation, and eventual apoptosis all implicated in disease pathogenesis (Kuang et al., 2023; Evangelho et al., 2019; Dammak et al., 2021). The role that biomechanical strain plays in regulating these processes has been heavily theorized (Burgoyne et al., 2005), but the direct contributions of pathologic biomechanical strain to cellular dysfunction is an area of active investigation. A critical barrier to our understanding of ONH cellular pathophysiology is the relative inaccessibility of this pivotal tissue in the posterior globe. To overcome this barrier, *in vitro* model systems provide ideal tools to study the mechanobiology—broadly defined as the mechanistic processing of biomechanical strains—of isolated cells of the optic nerve. The overarching goal of this review is to discuss the overall considerations and implications of available model systems.

## Glaucomatous ONH biomechanical strains as they pertain to *in vitro* models

There is considerable research dedicated to characterizing the biomechanical environment of the ONH (Liu et al., 2025; Islam et al., 2024; Karimi et al., 2022; Korneva et al., 2023; Korneva et al., 2020; Czerpak et al., 2024; Yuan et al., 2025). A primary driver of biomechanical strain on the ONH is intraocular pressure (IOP), and many studies have employed computational model systems to simulate ONH biomechanical behavior while varying IOP within physiologic and pathologic ranges (Midgett, Quigley, and Nguyen, 2019; Coudrillier et al., 2012). Finite element modeling (FEM) studies routinely incorporate parameters such as eye-specific geometry, collagen fiber characteristics, anisotropy and viscoelasticity to recapitulate the complex geometry and material properties of the ONH; these models are used to predict the type, magnitude, and loading distribution of the biomechanical strains within the tissue (Islam et al., 2024). Limitations of such computational studies include assumptions regarding material and boundary conditions and imaging resolution and background noise (Chuangsuwanich et al., 2023). More recently, direct measurements of tissue deformation (Korneva et al., 2023; Korneva et al., 2020; Czerpak et al., 2024; Yuan et al., 2025) and additional techniques like digital volume correlation (DVC) and optical coherence tomography (OCT)-based imaging, have been used to hone these computational models. Inclusion of both experimental measurements and computational models to predict the properties and dynamics of the ONH biomechanical milieu is likely to generate a comprehensive picture of ONH injury during glaucoma. For the purpose of this review, we focus on the application of biomechanical strains as they pertain to *in vitro* glaucoma model systems, where controlled strain loading and cell-material manipulation are powerful tools to gain insight into ONH cellular mechanodysfunction.

The source of biomechanical strains on the ONH is broadly derived from the difference between the IOP anterior to the ONH, and the optic nerve tissue pressure (ONTP) posterior to the ONH, along with the contribution of supportive tissues including the peripapillary sclera and extraocular muscles (Hopkins et al., 2020; Ji et al., 2023). The summation of ONH biomechanical strains can be simply defined as the difference between the IOP anteriorly and ONTP posteriorly (denoted as the translaminar pressure, TLP) (Hopkins et al., 2020). Since the majority of glaucomas are associated with elevated IOP (Weinreb, Aung, and Medeiros, 2014), the field of ONH biomechanics has largely focused on modeling pathologic biomechanical strains induced by elevated IOP as compared to physiologic biomechanical strains from “normal” IOP (which is 10–22 mmHg in humans).

There are several key points to note when interpreting these studies. First, ONH susceptibility to IOP varies widely across populations (Colton and Ederer, 1980). Eyes with ocular hypertension have higher IOPs, but do not develop glaucomatous damage, and eyes with normal tension glaucoma develop glaucomatous damage despite “normal” IOPs. It has been hypothesized that an abnormal ONTP, resulting in higher TLP at normal IOPs, may contribute to this varied ONH susceptibility. Second, as glaucoma severity increases (i.e., with greater glaucomatous optic nerve damage), there are increased baseline biomechanical strains on the ONH at equivalent IOPs (when compared to healthy ONH) (Czerpak et al., 2024; Yuan et al., 2025). It has also been shown that the ONH in normal tension glaucoma is subjected to higher biomechanical strains at baseline, at least when the contributions of eye movements are taken into account (Chuangsuwanich et al., 2024). Third, it is accepted that IOP is the main driver of biomechanical strain on the ONH (Karimi et al., 2022), and it is important to note that IOP varies widely on a seconds-minutes timescale due to factors such as extraocular movements and blinking (Downs, 2025; Jasien et al., 2019; Markert et al., 2018). This occurs in both healthy and glaucomatous eyes, although it has been shown that the glaucomatous ONH is subjected to greater and more frequent of these transient IOP elevations when compared to healthy ONH (Turner et al., 2019). In a sense, the relative ability of cells within the ONH to withstand pathologic biomechanical forces from elevated IOP is a defining characteristic of ONH susceptibility in glaucoma.

Most analyses of ONH biomechanical forces have focused on studying the impact of biomechanical strains (i.e., tensile, compressive, and shear strains) on ONH tissues in human/primates (Czerpak et al., 2024; Yuan et al., 2025; Sigal and Ethier, 2009) or rodents (Korneva et al., 2023; Korneva et al., 2020; Nguyen et al., 2018). It is important to differentiate between species since the primate ONH contains a lamina cribrosa (LC) with LC collagen beams and robust scleral collagen that can withstand higher IOPs when compared to mouse ONH (Morrison, Cepurna Ying Guo, and Johnson, 2011; Albrecht May 2008). To define glaucomatous biomechanical strains, most studies have calculated the generated ONH biomechanical strains from elevated IOP (typically set between 40–50 mmHg in humans/primates and 30 mmHg in rodents) *versus* normal IOP (typically set at 10 mmHg) (Karimi et al., 2021; Korneva et al., 2020). A fourth important consideration when interpreting these studies is the tendency to discuss the ONH as a somewhat homogenous tissue; however, the biomechanical strains within the ONH can vary quite dramatically microregionally, and recent studies have attempted to specifically quantify this variability (Downs et al., 2009; Voorhees et al., 2017; Karimi et al., 2021; Czerpak et al., 2023).

After acknowledging these defining characteristics, the general consensus for *in vitro* applications of biomechanical strains is that glaucomatous ONH tissues experience a range of 3%–5% tensile, shear, and compressive biomechanical strains in both primates and rodents, with certain microregions in the human ONH likely experiencing higher 5%–10% biomechanical strains (Karimi et al., 2021). An oversimplification is that the majority of ONH tissue is predominantly subjected to tensile strains, while compressive strains are concentrated on the peripheral ONH, but again, this varies widely microregionally (Karimi et al., 2021; Czerpak et al., 2023). Conceptual understanding of the biomechanical strain profile of the ONH is necessary to properly utilize *in vitro* model systems to study cellular mechanobiology to these glaucomatous biomechanical strains.

## Cells within the ONH

End stage glaucoma is associated with fibrosis and excess extracellular matrix (ECM) deposition within the ONH (Hopkins et al., 2020; Hernandez, 2000; Hernandez and Pena, 1997; Hernandez and Ye, 1993). The primary cell types responsible for depositing ECM in the ONH are local astrocytes and LC cells, and the majority of *in vitro* analyses to date have focused on these two cell types (Strat et al., 2025; Li et al., 2022; Wallace and O'Brien, 2016). Despite their close proximity to neighboring astrocytes within the retina and optic nerve proper, astrocytes within the ONH are developmentally distinct, arising from specialized neuroepithelial cells within the optic disc progenitor zone (Tao and Zhang, 2014; Paisley and Kay, 2021; Bernstein et al., 2020). As such, these cells exhibit a unique phenotype, harboring combined expression of axon guidance molecules, and optic stalk and ventral retina markers (Tao and Zhang, 2014; Paisley and Kay, 2021). These cells also do not express aquaporins, unlike other neighboring astrocytes (Kimball et al., 2022). LC cells, on the other hand, are present in primates that contain an LC and are absent in rodents (Tovar-Vidales, Wordinger, and Clark, 2016). Much less is known about their developmental origins. They appear to be a fibroblast-like cell type that are discretely glial fibrillary acidic protein (GFAP)-negative and alpha smooth muscle actin (αSMA)-positive *in situ* (Tovar-Vidales, Wordinger, and Clark, 2016; Monavarfeshani et al., 2023). It is also thought that they are primarily responsible for secreting the dense collagenous beams that form the primate LC (Wallace and O'Brien, 2016).

The source of these particular ONH cells begs consideration. ONH astrocytes are typically cultured directly either from human donor eye globes (Lopez, Clark, and Tovar-Vidales, 2020; Hernandez, Igoe, and Neufeld, 1988) or from mouse or rat explant ONH tissue (Kirschner et al., 2021; Strat et al., 2022; Zhao et al., 2021; Hariani et al., 2024). LC cells are similarly cultured from human donor eye globes (Lopez, Clark, and Tovar-Vidales, 2020; Lopez et al., 2021; Hernandez, Igoe, and Neufeld, 1988). Interestingly, both human ONH astrocytes and LC cells are cultured from the same explant tissue; in contrast to LC cells, ONH astrocytes survive intentional serum deprivation (Lopez, Clark, and Tovar-Vidales, 2020). It is unknown if these harsh cell culture conditions used to isolate human ONH astrocytes may select for more reactive cells, and thereby bias experimental readouts. Human donor-derived cells likely better mimic human glaucoma pathophysiology, but caveats include that human donor tissue is often acquired ∼24 h after death, and thus may affect primary cell isolations. On the other hand, rodent ONH astrocytes are easily cultured from explant tissue, but are often derived from young mice (∼6–8 weeks) (Strat et al., 2022; Kirschner et al., 2021). For both culture sources, minimization of the number of cell passages may better maintain cellular identity (Bang et al., 2021; Guler, Krzysko, and Wolfrum, 2021).

Many studies have used bioreactors to apply tensile or compressive biomechanical strains to ONH astrocyte or LC cell cultures, and these studies support that biomechanical strains directly induce ECM deposition and fibrosis (Quill et al., 2011; Kirwan et al., 2005; Strat et al., 2025; Rogers et al., 2012). Additionally, *in vitro* analyses support a direct relationship between biomechanical strain application and ONH astrocyte inflammation and metabolic insufficiency (Strat et al., 2025; Pappenhagen et al., 2022). Since ONH astrocytes provide pivotal neurotrophic support to retinal ganglion cell (RGC) axons (Ribeiro et al., 2022; Bernstein et al., 2020), these more recent experiments point to new possible mechanisms driving cellular dysfunction in glaucoma.

Much less is known about the mechanobiology of the other cell types within the ONH, which include microglia, endothelial cells, pericytes, and – most relevant to glaucoma – RGC axons (Hernandez, 2000; Neufeld, Hernandez, and Gonzalez, 1997). Microglia within the central nervous system are known to respond directly to biomechanical strain (Proces et al., 2024; Shaughness et al., 2023), and a few studies have investigated mechanisms by which retinal microglia respond to osmotic stress (Redmon et al., 2021) and to tissue stiffness during development (Dudiki et al., 2020). However, no studies to date have investigated how tensile or compressive biomechanical strain regulates microglial behavior within the ONH. Further investigation into the mechanisms regulating ONH microglial, vascular, and RGC axonal mechanobiology is necessary to address this major gap in our understanding of glaucoma pathophysiology.

## 2D vs. 3D culture systems

**FIGURE 1:**
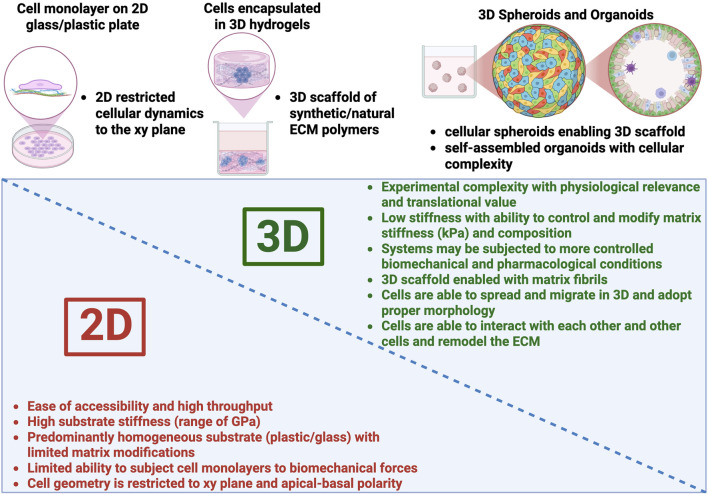
Characteristics of 2D vs. 3D *in vitro* cell culture model systems. Illustration created with BioRender®.

To study biomechanical strain-induced cellular dysfunction, traditional two-dimensional (2D) cell cultures have been widely used due to their simplicity and cost-effectiveness (Vernazza et al., 2019). 2D cultures of ONH cells have been used to investigate fundamental aspects of glial biology, including changes pertaining to morphology, gene expression and reactivity, as well as glaucoma-related biomechanical strains (Pitha et al., 2024; Vroemen et al., 2019). However, it is important to consider the limitations of these 2D stretch systems. Generally, these systems rely on cell monolayer cultures on flexible membranes, which are stiffer (∼10 kPa) than native neural tissue (Jensen and Teng, 2020; Budday et al., 2015; Budday et al., 2017). Additionally, the flat substrate can impose geometric constraints on the cells, resulting in altered morphology (Kirschner et al., 2021) and, in the case of ONH glia, a potentially reactive phenotype, thereby limiting their physiological relevance (Caliari and Burdick, 2016; Jensen and Teng, 2020). Overall, these 2D cultures fail to replicate the complex three-dimensional (3D) architecture and cell-cell and cell-matrix interactions present in native tissues (Figure 1).

An alternate option to 2D culture systems is 3D cell-encapsulated *in vitro* systems that attempt to replicate the structural and biochemical complexity of the *in vivo* microenvironment (Figure 1) (Urzi et al., 2023; Fang and Eglen, 2017). Among these, viscoelastic hydrogels (i.e., water-swollen networks of natural or synthetic polymers) have emerged as a promising platform to offer a more physiologically relevant scaffold for modeling cell behavior (Zhang and Khademhosseini, 2017; Zhang et al., 2015; Lee and Mooney, 2001). Hydrogels are hydrated polymer networks typically used in tissue engineering, stem cell research, tumor modeling, and drug discovery (Davari et al., 2022; Drury and Mooney, 2003; Liu et al., 2023; Seidlits et al., 2010; Fang and Eglen, 2017).

Hydrogels consisting of ECM components have emerged as a promising platform to bridge the gap between *in vitro* and *in vivo* models (Urzi et al., 2023; Fang and Eglen, 2017; Placone et al., 2015; Li et al., 2021). These constructs can be generated from natural components such as collagen and hyaluronic acid (HA), and may also incorporate synthetic polymers like polyethylene glycol (Geckil et al., 2010; Placone et al., 2015). Matrigel, a protein mixture extracted from mouse sarcoma cells, has been used to study ocular cell physiology (Bouchemi et al., 2017); however, the batch-to-batch variability in ECM composition is a significant drawback (Caliari and Burdick, 2016). In addition to collagen, gelatin methacrylate (GelMA) is a widely used photocrosslinkable version of gelatin, and chitosan is a natural polysaccharide derived from chitin (Roegiers et al., 2025; Dogan et al., 2023; Zhao et al., 2023; Ahmadi et al., 2015). Both components are recognized for their cytocompatibility and biodegradability (Lazaridou, Bikiaris, and Lamprou, 2022). GelMA has been used to study retinal cell communication (Qureshi et al., 2025), and scaffold-combinations of chitosan and GelMA can guide axonal elongation in peripheral nerve tissue engineering studies (Yu, Zhang, and Li, 2020), but neither polymer has yet been used to study ONH cell behavior.

Importantly, the biomechanical properties of these hydrogels can be regulated by choice of crosslinker, construct swelling characteristics, and the exact concentration and composition of ECM components incorporated (Blache et al., 2022; Khare et al., 2024; Morton et al., 2023; Fraser et al., 2022; Gupta and Shivakumar 2012). For example, the structural properties and energy potential of hydrogel crosslinkers can impact the viscosity level of the ECM network (Khare et al., 2024). Moreover, in studies of photocrosslinkable ECM hydrogels, the choice of photoinitiator can affect the stiffness properties of the matrix. Multiple studies, including ECM hydrogels that encapsulate ocular cells, have successfully used photoinitiatiors like LAP (lithium phenyl-2,4,6-trimethylbenzoylphosphinate; at a concentration of 0.05%–2.0% (w/v)) (Singh et al., 2025), Irgacure® 2959 (4-(2-hydroxyethoxy) phenyl-(2-propyl) ketone; at a concentration of 0.5% (w/v)) (Li et al., 2021) or riboflavin (vitamin B2; at a concentration of 0.025% (w/v)) (Strat et al., 2022). Depending on the cell type and ECM hydrogel composition, an increased concentration of these photoinitiators may affect cell viability within these constructs. Riboflavin is an exception to this rule as it is used clinically as a cytoprotective collagen crosslinker for treatment of ocular keratectasia (Wollensak, Spoerl, and Seiler, 2003). Lastly, the swelling properties of the ECM hydrogels can also influence cell behavior. This property is primarily modulated by the size of the pores, and microstructure of the matrix network (Gupta and Shivakumar 2012; Fraser et al., 2022).

By carefully considering these structural properties of ECM hydrogel model systems, these 3D scaffolds enable encapsulated cells to adopt physiologically relevant morphologies, gene expression profiles, and functional behaviors not typically observed with 2D cultures (Brevini, Pennarossa, and Gandolfi, 2020; Strat et al., 2022). This is especially critical in studies of astrocytes whose stellate morphology and function are tightly regulated by the surrounding microenvironment. *In situ*, these astrocytes interact closely with ECM proteins such as collagens, HA, proteoglycans, tenascins, and laminins (Tewari et al., 2022; Wiese, Karus, and Faissner, 2012). Recreating an ECM scaffold in 3D culture promotes astrocyte process interaction with surrounding ECM proteins, thereby allowing development of stellate morphology and secretion of additional ECM proteins into this scaffold (Placone et al., 2015; Strat et al., 2022). As such, these 3D ECM hydrogel models are beneficial to model changes in astrocyte morphology and ECM interactions that drive transition to a pathological phenotype.

A few 3D systems have been developed to emulate the biomechanical and biochemical properties of neural tissue (Georges et al., 2006; Castillo Ransanz et al., 2022). Since HA and collagen are abundantly present in neural tissue, 3D ECM hydrogels incorporating such natural components in various combinations (i.e., HA, collagen, collagen + HA, and HA + basal lamina proteins) have shown promise in supporting astrocytes (Placone et al., 2015; Koss et al., 2017). Specifically, encapsulated cortical astrocytes in collagen + HA showed a highly branched, star-shaped morphology with low reactivity (Placone et al., 2015). An additional benefit of 3D systems is that encapsulated cells can be subjected to *both* tensile biomechanical strain (Strat et al., 2024) and compressive biomechanical strain (Strat et al., 2025), thereby advancing our understanding of biomechanical strain-induced ONH cellular dysfunction. Future studies may attempt to mimic the ECM beam structure of primates by micropatterning the ECM hydrogel, or bioprinting a model LC (Shittu et al., 2023). Shortcomings of these 3D systems predominantly include limitations with live-cell imaging and cellular/biomechanical assays, since cells must be released from their surrounding ECM network prior to analysis. Ultimately, careful understanding of the benefits/limitations of each culture system is necessary to investigate ONH cellular mechanobiology *in vitro*.

## Experimental parameters of glaucomatous biomechanical strain application

The main variables for *in vitro* glaucoma models include the selection of bioreactor to apply biomechanical strain, and the magnitude, axis, and frequency of biomechanical strain. The most common commercially available bioreactor is the FlexCell 2D system (Dyment et al., 2020). Such 2D tensile bioreactors subject cells that are seeded on a flexible membrane to vacuum suction or stretch-inducing chambers to apply tensile strains. A variation of the standard 2D tensile bioreactor permits stretching of 3D hydrogels through modifications such as a circumferential foam mesh that allow the hydrogel to affix to the chamber walls (Dyment et al., 2020). In contrast, compressive bioreactors typically use a platen to compress 3D constructs vertically (McDermott et al., 2019). In addition to increased compressive/tensile biomechanical strains, glaucoma severity is also associated with ECM stiffening (Hopkins et al., 2020). As such, an alternate method to subject ONH cells to glaucomatous biomechanical strains is to increase substrate stiffness. A few studies have seeded LC cells atop either silicone membranes or polyacrylamide gels of different stiffnesses to examine the effect of substrate stiffness on cellular behavior (Liu et al., 2018; Murphy et al., 2022; Irnaten, Gaynor, and O'Brien, 2024; Shi and Janmey, 2023).

For both tensile and compressive biomechanical strain application, most *in vitro* studies subject cells to 3%–5% superimposed biomechanical strain to mimic the pathologic biomechanical strains observed in glaucoma, as discussed earlier (Strat et al., 2025; Korneva et al., 2020). Of course, even at 0% applied biomechanical strain, each cell has a baseline level of biomechanical strain due to its adhesion with its substrate in 2D systems (Janmey, Hinz, and McCulloch, 2021), or its interaction with the surrounding ECM in 3D systems (Rocha et al., 2015). Similarly, at physiologic (normal) IOPs, ONH cells *in situ* are exposed to baseline levels of biomechanical strain due to their surrounding ECM and baseline IOP (Sigal and Ethier, 2009). These baseline biomechanical strains then increase during glaucomatous conditions; therefore, bioreactors used to mimic glaucomatous biomechanical strain levels intend to mimic the changes in glaucomatous biomechanical strains from baseline.

When subjecting ONH cells to compression, the axis of biomechanical strain is singular; these bioreactors typically compress encapsulated cells in a superior to inferior direction (Strat et al., 2025; Mulvihill et al., 2018; McDermott et al., 2019; McDermott et al., 2021). When applying tension, however, cells may be subjected to a single axis of biomechanical strain (uniaxial) or multiple axes of biomechanical strain (multiaxial or equibiaxial) (Vande Geest et al., 2004). Regardless of the primary biomechanical strain axis, all bioreactors will also subject cells to secondary biomechanical strains. For example, cells encapsulated within a 3D hydrogel and subjected to compressive strains likely additionally experience tensile strains (greater in the periphery of the hydrogel if its boundaries are not physically confined), and some amount of shear strain. The relative contribution of each of these biomechanical strains will vary based on the applied primary biomechanical strain and the viscoelastic properties of the cells/hydrogel.

The frequency of applied biomechanical strain is most often set at 1 Hz, and typically oscillates between 0% and target (i.e., 3%–5%) compressive or tensile biomechanical strain. The rationale cited for this particular frequency is often that the ocular pulse rate in humans is directly linked to the heart rate, which is roughly 60 beats/minute (i.e., 1 Hz). However, 1Hz frequency is often also used to study rodent-derived cells (Mulvihill et al., 2018; Strat et al., 2025; Pappenhagen et al., 2022), but rodents have a higher heart rate (Janssen et al., 2016). Thus, a higher applied frequency may be more biologically relevant. Additionally, the biomechanical strains derived from ocular pulse amplitude do not oscillate between 0%–5% biomechanical strain (Jin et al., 2020). Instead, it may be more accurate to increase static tensile or compressive biomechanical strain to 5% (to mimic glaucomatous IOP elevation) and to superimpose 1%–2% cyclic biomechanical strain to mimic the variations from the ocular pulse amplitude. Lastly, all studies investigating ONH cell mechanobiology to date have subjected cells to a regular cyclic frequency of biomechanical strain (Strat et al., 2025; Pappenhagen et al., 2022; Quill et al., 2011). The impact of irregular frequencies of biomechanical strain (such as would be seen during transient IOP elevations) has not yet been investigated in this context. This may be an interesting avenue for future research, since irregular frequencies of biomechanical strain have been shown to affect cell behavior differently from regular cyclic frequencies (Bartolak-Suki et al., 2015; Arold, Bartolak-Suki, and Suki, 2009).

## Conclusions and future directions

**FIGURE 2:**
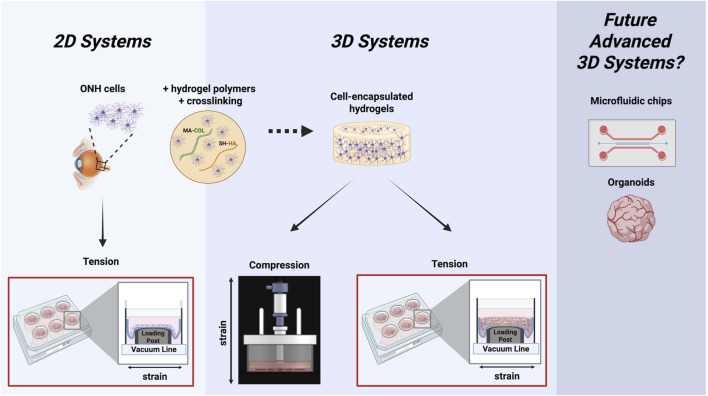
*In vitro* model systems to study ONH cellular mechanobiology. 2D model systems include simplified seeding of ONH cells on a flexible membrane to apply tension. Newer 3D model systems encapsulate ONH cells within functionalized ECM polymers to permit application of compressive or tensile biomechanical strain. Future systems may overcome limitations of existing models by developing microfluidic chips to permit biomechanical strain application to ONH cell-encapsulated 3D hydrogels or even develop ONH organoids to better recapitulate the native communication between neurons, ONH glia, and vasculature. Illustration created using BioRender®.

Mechanobiology is broadly defined as the mechanisms by which cells sense, transduce, and respond to information about the surrounding biomechanical environment (Eyckmans et al., 2011). Pathologic biomechanical strains on the ONH have long been implicated in disease pathogenesis in glaucoma (Burgoyne et al., 2005), but the mechanobiology of the cells within this region is much less understood. *In vitro* model systems designed to apply glaucoma-like pathologic biomechanical strains to isolated cells of the ONH can serve as useful tools to address this knowledge gap (Figure 2). To date, the majority of studies that have investigated such mechanisms involve cultured ONH astrocytes or LC cells seeded on a stretchable membrane and subjected to glaucomatous tensile strains (Pappenhagen et al., 2022; Kirwan et al., 2005). Recent work has attempted to advance these model systems by encapsulating ONH cells within a 3D ECM hydrogel, to allow for better assessment of cellular morphology and cell-ECM interactions (Strat et al., 2025). These 3D model systems act as a bridge between traditional 2D cell culture systems and the complex *in vivo* environment (Urzi et al., 2023; Ho et al., 2022; Zoller et al., 2025). As such, they permit detailed analyses of cellular mechanisms that can then be tested in relevant *in vivo* glaucoma models. Persistent limitations of currently available model systems include difficulties with live-cell imaging and the absence of several important ONH cell types. Future models could incorporate microfluidic chips to apply biomechanical strain to 3D cell-encapsulated hydrogels or even develop ONH organoids to better recapitulate the native communication between RGCs, ONH glia, and vasculature.

The cells within the ONH are inherently mechanosensitive (Choi, Sun, and Jakobs, 2015; Wan et al., 2023; Irnaten, Gaynor, and O'Brien, 2024; Liu et al., 2018), and the field is beginning to use these *in vitro* model systems to elucidate mechanisms regulating ONH cell sensation and transduction of glaucomatous biomechanical strains. Mechanosensitive channels, integrins, cytoskeletal proteins, and yes-associated protein signaling have been identified as key transducers of increased biomechanical strain in ONH astrocytes and LC cells (Irnaten, Gaynor, and O'Brien, 2024; Murphy et al., 2022; Strat et al., 2024). Future *in vivo* studies can test the therapeutic potential of targeting these pathways. Major gaps in our understanding of ONH mechanobiology remain; mainly, the mechanobiology of several ONH cell types (RGC axons, endothelial cells, pericytes, microglia) have yet to be investigated, and mechanisms of intracellular crosstalk in response to biomechanical strain are largely unknown. As such, there is a need for continued advancement of existing *in vitro* ONH model systems to incorporate multiple ONH cell types, to more faithfully recapitulate the LC architecture, and to study the effects of glial and endothelial cell activation on neuronal health. Ultimately, harnessing the diverse *in vitro* systems available to study ONH cellular mechanobiology will continue to elucidate the contributions of pathologic biomechanical strains to ONH dysfunction in glaucoma.

## Abbreviations

αSMA, alpha-smooth muscle actin; 2D, two dimensional; 3D, three dimensional; DVC, digital volume correction; ECM, extracellular matrix; FEM, finite element modeling; GFAP, glial fibrillary acidic protein; HA, hyaluronic acid; IOP, intraocular pressure; LC, lamina cribrosa; OCT, optical coherence tomography; ONH, optic nerve head; ONTP, optic nerve tissue pressure; RGC, retinal ganglion cell; TLP, translaminar pressure.
